# Combined Nabpaclitaxel pressurized intraPeritoneal aerosol chemotherapy with systemic Nabpaclitaxel-Gemcitabine chemotherapy for pancreatic cancer peritoneal metastases: protocol of single-arm, open-label, phase II trial (Nab-PIPAC trial)

**DOI:** 10.1515/pp-2024-0010

**Published:** 2024-11-06

**Authors:** Andrea Di Giorgio, Federica Ferracci, Cinzia Bagalà, Carmine Carbone, Lisa Salvatore, Antonia Strippoli, Miriam Attalla El Halabieh, Carlo Abatini, Sergio Alfieri, Fabio Pacelli, Giampaolo Tortora

**Affiliations:** Surgical Unit of Peritoneum and Retroperitoneum, Fondazione Policlinico Universitario A. Gemelli IRCCS, Rome, Italy; Comprehensive Cancer Center, Fondazione Policlinico Universitario A. Gemelli IRCCS, Catholic University of the Sacred Heart, Rome, Italy; Surgical Unit of Digestive Surgery, Fondazione Policlinico Universitario A. Gemelli IRCCS, Rome, Italy; Catholic University of the Sacred Heart, Rome, Italy; Department of Medical and Surgical Science, Fondazione Policlinico Universitario A. Gemelli IRCCS, Rome, Italy

**Keywords:** peritoneal metastasis, pancreatic cancer, PIPAC, bidirectional chemotherapy, combined chemotherapy, locoregional chemotherapy

## Abstract

**Objectives:**

Current therapies show limited efficacy against peritoneal metastases (PM) from pancreatic cancer. Pressurized intra-peritoneal aerosol chemotherapy (PIPAC) has emerged as a novel intraperitoneal drug delivery method. Recently, a dose-escalation study identified the safe dose of Nabpaclitaxel for PIPAC administration, an ideal intraperitoneal chemotherapy agent against pancreatic cancer. Combining systemic NabPaclitaxel-Gemcitabine with NabPaclitaxel-PIPAC may enhance disease control in pancreatic cancer patients with PM.

**Methods:**

The Nab-PIPAC trial is a single-center, prospective, open-label, phase II study (ClinicalTrials.gov identifier: NCT05371223). Its primary goal is to evaluate the antitumor activity of the combined treatment based on Disease Control Rate (DCR) using RECISTv.1.1 criteria. Secondary objectives include feasibility, safety, pathological response, progression-free and overall survival, nutritional status, quality of life, pharmacokinetics of NabPaclitaxel-PIPAC, and PM molecular evolution via translational research. The treatment protocol consists of three courses, each with two cycles of intravenous NabPaclitaxel-Gemcitabine and one cycle of NabPaclitaxel-PIPAC, with standard metastatic pancreatic cancer doses for the former and 112.5 mg/m^2^ for the latter. Sample size follows Simon’s two-stage design: 12 patients in stage one and 26 in stage two (80 % power, 0.1 alpha).

**Results:**

Partial results will be available after first stage enrollment.

**Conclusions:**

This trial aims to determine the antitumor efficacy and safety of combining NabPaclitaxel-PIPAC with systemic NabPaclitaxel-Gemcitabine in pancreatic cancer patients with PM.

## Background

Pancreatic cancer is the fourth leading cause of cancer mortality in the West despite its relatively low incidence, with 43.500 estimated deaths in 2022 in the European Union [1].

Prognosis has been consistently poor over the last decades [2], with a 5-year survival rate of only 8 % following initial diagnosis [3]. Contrary to other malignant diseases, few advances have been made and the observed trends in mortality rates remained stable or slightly increased [4].

Surgery represents the only treatment option with curative potential but only 15 % of patients can undergo primary tumor resection [5]. Most of patients have already developed at time of diagnosis a locally advanced or metastatic disease and are treated with systemic chemotherapy or palliative care.

The peritoneum is the second most common site of metastasis after the liver. Indeed, peritoneal dissemination is a major concern in pancreatic cancer affecting at least 1 in every 8 patients at the time of diagnosis [6] and ultimately developing in up to 50 % of cases [7]. These patients are striving for new treatment options effectively addressing peritoneal disease as systemic chemotherapy hardly reaches peritoneal implants, resulting in very poor outcomes. Compared to systemic chemotherapy, intraperitoneal chemotherapy provides higher drug concentrations directly targeting tumor nodules with less systemic exposure and might be more advantageous in treating peritoneal disease. The results of recent Asian trials on the combination of systemic and port-based intraperitoneal chemotherapy are encouraging 8], [9], [10.

Recently, pressurized intraperitoneal aerosol chemotherapy (PIPAC) emerged as a novel intraperitoneal drug-delivery system of low-dose chemotherapy as a pressurized aerosol. It combines high intraperitoneal concentration, low systemic concentration and toxicity with the homogeneous intraperitoneal distribution and deeper tissue penetration of aerosol. Data from several non-comparative clinical studies with various intraperitoneal chemotherapy drugs suggest that PIPAC is a safe, feasible, and well-tolerated treatment showing good preliminary response rates on peritoneal metastases (PM) of various origins. Furthermore, due to its repeatable, minimally-invasive nature, PIPAC was successfully combined with current systemic chemotherapy regimens [11, 12].

So far, prospective series have reported promising feasibility, safety and antitumor activity data on PIPAC with cisplatin/doxorubicin or oxaliplatin for pancreatic cancer peritoneal metastases 13], [14], [15], [16], [17. However, no phase-II PIPAC trials addressed to pancreatic cancer peritoneal metastases have been conducted.

Based on preclinical and clinical data, Nabpaclitaxel is an ideal candidate for intraperitoneal chemotherapy and is highly active on pancreatic cancer cells 18], [19], [20.

Recently, a phase I study (NCT03304210) explored its use with PIPAC resulting well tolerated, with a favorable PK profile and promising anticancer activity in patients with PM. This study identified the dose to safely start a phase-II trial [21]. A phase IB trial from Switzerland is ongoing to determine the MDT of IP Nab-paclitaxel administered by PIPAC in concomitant with IP Cisplatin and to access the safety and tolerability of this combined PIPAC treatment [22].

We designed the present study to test Nabpaclitaxel-PIPAC in combination with systemic Nabpaclitaxel-Gemcitabine for the treatment of pancreatic cancer PM.

## Patients and methods

The Nab-PIPAC trial is a monocentric prospective, open-label, phase II study assessing the combined treatment of endovenous Nabpaclitaxel-Gemcitabine and intraperitoneal Nabpaclitaxel administered through PIPAC for pancreatic cancer peritoneal metastasis.

### Primary objective

The main objective is to evaluate the antitumor activity of this combined chemotherapy. The primary endpoint is the disease control rate (DCR) defined as the combined incidence of complete response (CR), partial response (PR), and stable disease (SD) according to the RECIST v. 1.1 criteria.

### Secondary objectives

The secondary objectives include the assessment of feasibility, safety, pathological tumor response, progression-free and overall survival, and quality of life of the combined treatment. In addition, the pharmacokinetics of Nab-PIPAC, the nutritional status assessment and a translational study on the mutational, transcriptomic, and immune cells profiles have been planned. Table 1 shows the endpoints considered for the evaluation of the secondary objectives.

**Table 1:** Secondary endpoints.

Objectives	Endpoints
Feasibility	– Number of patients unable to undergo six cycles of systemic chemotherapy combined with three PIPAC cycles and reasons for discontinuation – Number of patients who discontinued or postponed beyond 10 days the scheduled standard systemic chemotherapy after each PIPAC cycle – Number of patients that reduce the dose of systemic chemotherapy during the study – Number of patients that reduce the dose of PIPAC drug during the study
Safety	– Number of patients with major toxicity, defined as grade ≥3 according to CTCAE v5.0 during the on-study evaluation phase and up to four weeks after the last chemotherapy administration – Number of patients with minor toxicity, defined as grade ≤2 according to CTCAE v5.0 during the treatment period and up to 4 weeks after the last combined course – Number of patients with major and minor postoperative complications, defined as grade ≥3 and grade ≤2 according to Clavien-Dindo, respectively, during the treatment period and up to 4 weeks after the last PIPAC procedure – Hospital stays, which is defined as the number of days between PIPAC and initial discharge – Number of readmissions, defined as any hospital admission after discharge, up to four weeks after the last PIPAC procedure
Antitumor activity	– Pathological tumor response, based on the review of peritoneal biopsies collected during each PIPAC, performed by a pathologist blinded to clinical outcomes using the PRGS. A patient will be considered a responder if any reduction in the PRGS during subsequent biopsies will be recorded – Macroscopic tumor response, based on PCI and ascites volume recorded during each PIPAC – Biochemical tumor response, based on tumor markers (CEA, Ca 19.9, Ca 125) measured at different time points – Quality of life of the population will be assessed through the potential changes in QoL scores extracted from the questionnaire QLQ-C30 at different time points – PFS defined as the time between treatment start and one of the following events, whichever comes first: – Radiologic progression based on RECIST criteria v. 1.1 – Clinical progression (i.e., bowel occlusion, inability to oral feeding, refractory ascites) – Death – The OS is defined as the time between treatment start and death

PIPAC, Pressurized Intraperitoneal Aerosol Chemotherapy; CTCAE, Common Terminology Criteria for Adverse Events; PRGS, Peritoneal Regression Grading Score; PCI, Peritoneal Cancer Index; QoL, quality of live; QLQ-30, Quality of Life Questionnaire; PFS, Progression-Free Survival; OS, Overall Survival.

### Study assessment and time points

Each patient will be treated to complete three combined courses of endovenous Nabpaclitaxel-Gemcitabine chemotherapy and Nab-PIPAC. Patients will undergo disease response assessment at each combined course.

The study period will include the screening phase, the study treatment phase (until 30 days after the last dose of Nabpaclitaxel- Gemcitabine or last PIPAC) and the follow-up period. The treatment phase will start with the administration of systemic Nabpaclitaxel-Gemcitabine, for two cycles, according to the standard protocol. Nabpaclitaxel-PIPAC will be started 10–13 days after the last dose of the second cycle of systemic chemotherapy and repeated once every two cycles of systemic Nabpaclitaxel-Gemcitabine. Each patient will have three repetitive combined courses.

**Figure 1:**
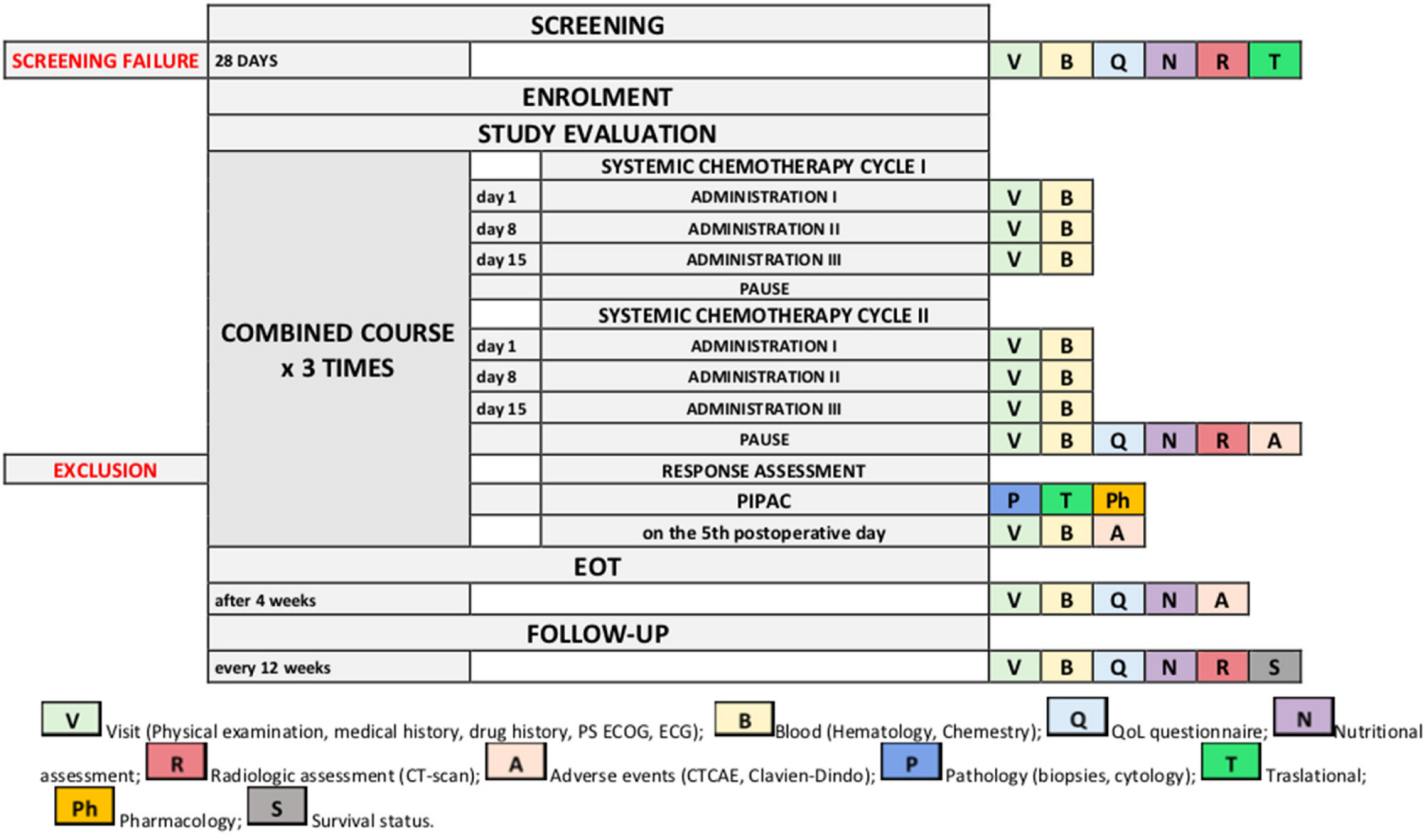
Flow-chart of the study with time-points evaluations.

Figure 1 shows the timing of the assessments performed during the study period.

Radiological tumor response assessment according to RECIST is performed after every two cycles of systemic chemotherapy just before each PIPAC. Patients who progressed will not proceed to PIPAC and drop out the protocol. Study treatment is discontinued in case of physician-determined disease progression, unacceptable toxicity or physician’s or patient’s decision to discontinue participation. Study treatment ends after the third PIPAC, regardless of response to therapy.

**Figure 2:**
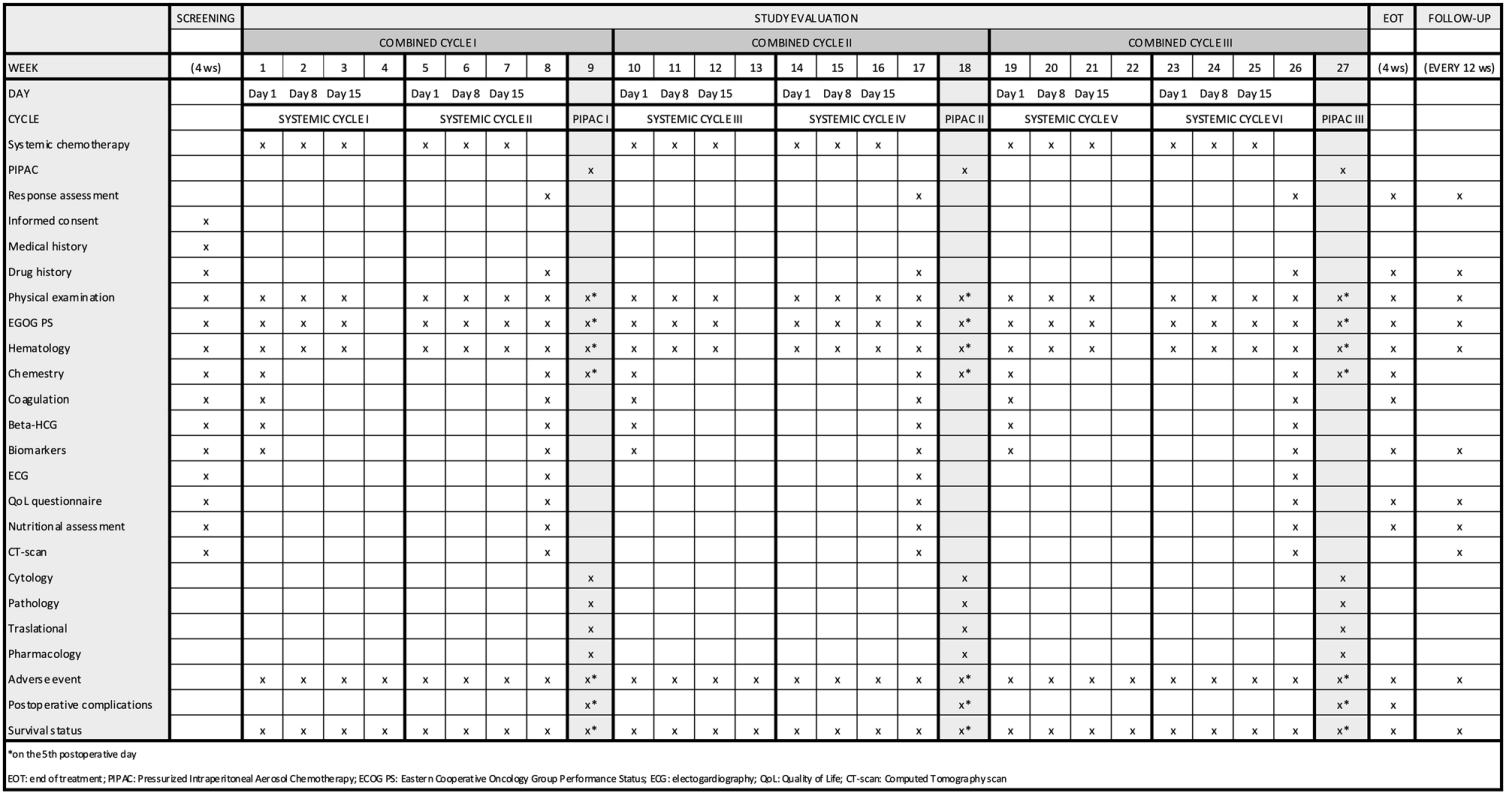
Study procedures chart.

The schedule of enrolment, interventions and assessments is shown in Figure 2.

### Eligibility criteria

Patients with pancreatic cancer peritoneal metastasis will be evaluated for eligibility to the study by the multidisciplinary tumor board (MTB) based on the screening assessment and the following criteria. Inclusion and exclusion criteria are reported in Table 2.

**Table 2:** Inclusion and exclusion criteria for Nab-PIPAC trial.

Inclusion criteria	Exclusion criteria
Pancreatic cancer with peritoneal metastases determined based on abdominal CT or MR and/or diagnostic laparotomy or laparoscopy	-urgical or medical contraindications to laparoscopy
Histological or cytological proof of pancreatic cancer	Advanced metastatic systemic disease with clinical deterioration
ECOG performance status 0 or 1	Patients with extra-abdominal tumor spread
Life expectancy of at least 3 months	Patients with a germline or somatic pathogenic variant involving an HRR-related gene
Absolute neutrophil count ≥1,500 cell/mm^3^	Symptoms of gastrointestinal occlusion and total parenteral nutritional support
Platelets ≥100,000 cell/mm^3^	-atients defined as “refractory” to previous systemic treatment with Nabpaclitaxel and Gemcitabine administered for locally advanced pancreatic cancer
Hemoglobin ≥9 g/dL	-nown hypersensitivity reaction to drugs chemically related to Nabpaclitaxel, Gemcitabine and their excipients
Adequate renal function	History of severe and unexpected reactions to Nabpaclitaxel or Gemcitabine derivates
Resolution of all toxic effects of prior therapies or surgical procedures to grade ≤1 (except alopecia and peripheral neuropathy)	
In absence of liver metastases, ALT and AST ≤2.5 × ULN. With liver metastases, ALT and AST <5 × ULN, total bilirubin ≤ULN, or total bilirubin 1.5 × ULN with direct bilirubin ≤ULN of the laboratory in subjects with documented Gilbert’s syndrome	
Age≥18 years	
Informed consent	

CT, Computed Tomography; MR, Magnetic Resonance; ECOG, Eastern Cooperative Oncology Group; ALT, alanine aminotransferase; AST, aspartate aminotransferase; ULN, upper limit of normal; HRR, Homologous Recombination Repair; PM, peritoneal metastases.

### Study treatments

**Figure 3:**
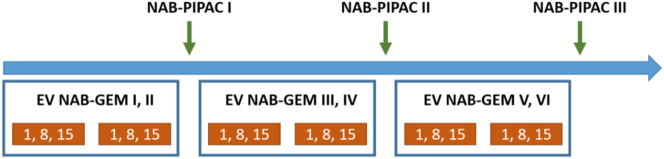
Timeline of combined systemic Nabpaclitaxel-Gemcitabine and Nab-PIPAC.

Each patient is scheduled for three combined courses of endovenous chemotherapy Nabpaclitaxel-Gemcitabine 125/1,000 mg/m^2^ and Nabpaclitaxel-PIPAC 112.5 mg/m^2^ (Figure 3). Each combined course lasts nine weeks and is constituted by two 28-day cycles of systemic chemotherapy (three administrations per cycle: days 1, 8 and 15) and one cycle of PIPAC administered within 10–13 days from the last administration of the second systemic cycle.

The PIPAC procedure will be carried out according to the standard technique previously reported [23] and briefly described. After each PIPAC, systemic chemotherapy was resumed in 7–10 days. Hence, each patient will receive a total of VI cycles of systemic chemotherapy and III PIPAC administrations. The study treatment ends after the third PIPAC in all patients.

### Statistical analysis

Concerning the analysis of the primary endpoint, the DCR, all time-points responses observed while on study treatment and during the EOT visit will be included in the derivation. The ratio of the rate and its 95 % CI will be presented.

Safety, feasibility, and QoL endpoints will be reported by descriptive statistics. PFS and OS will be presented by median times and associated 95 % CI as well as by survival curves using the Kaplan-Meier method. Continuous data will be summarized using the number of available data, mean, standard deviation. Categorical data will be summarized using the number and percentage of patients.

### Sample size

A total of 38 patients affected by pancreatic carcinoma with peritoneal metastases undergoing combined systemic and intraperitoneal chemotherapy will be enrolled.

Simon’s two-stage design was used to calculate the sample size for this trial [24]. With a Power of 80 %, P0=40 %, and P1=60 %, 12 patients will be enrolled in the first stage; with 6 or more patients experiencing CR/PR/SD, at this stage, another 26 patients will be enrolled in the second stage. The study will be considered positive with an alpha error=0.1, whether 19 or more patients will experience CR/PR/SD. The planned duration of the study is 36 months.

### Data collection and management

Data will be collected using an electronic case report form (CRF).

The sponsor maintains confidentiality standards by assigning a patient identification number.

On all trial-specific documents, other than the signed consent, the participant will be referred to by the trial participant number/code.

### Monitoring

#### Data monitoring

According to the international conference on harmonisation good clinical practice (ICH GCP), the monitoring team must check the CRF entries against the source documents, except for the pre-identified source data directly recorded in the CRF. The informed consent form will include a statement by which the patient allows the Sponsor’s duly authorized personnel, the Ethics Committee (IRB/IEC), and the regulatory authorities to have direct access to original medical records, which support the data on the CRFs (e.g., patient’s medical file, appointment books, original laboratory records, etc.). These personnel, bound by professional secrecy, must maintain the confidentiality of all personal identity or personal medical information (according to confidentiality and personal data protection rules).

#### Safety reporting

The collection, assessment and presentation of safety reports will be carried out in accordance with the detailed guidance on the collection, verification and presentation of adverse event/reaction reports arising from clinical trials on medicinal products for human use (‘CT-3’).

Patients will be carefully monitored for any AE occurring during the trial conduct. Such monitoring also includes clinical laboratory tests. AEs will be assessed in terms of their seriousness, severity, and causal relation to the study treatment. Safety reporting to study investigators, ECs, competent authorities will then follow in accordance with the results of such assessment.

#### Ethics approval, consent to participate, and dissemination

The trial will be conducted in accordance with the ethical principles set out in the Declaration of Helsinki and are consistent with ICH/Good Clinical Practice and regulatory requirements for participant data protection.

Before participating in the investigation, participants will receive comprehensive information concerning the trial through both verbal communication and a written consent form. Participants will be duly informed of their prerogative to discontinue their involvement in the trial at any given juncture.

The study received the approval of the Italian drug agency (AIFA) (Approval Code: 2021-002539-51 SC 22540); Date of approval: 18/10/2021.

Institutional Review Board approval of the Ethical Committee of the Fondazione Policlinico Universitario Agostino Gemelli was obtained. Institutional Review Board number: ID CE 4368; Date of approval: 07/01/2022.

During the clinical trial, any amendment or modification to the clinical trial protocol should be submitted to the IRB/IEC before implementation, unless the change is necessary to eliminate an immediate hazard to the patients, in which case the IRB/IEC should be informed as soon as possible.

Property of data is of Fondazione Policlinico Universitario Agostino Gemelli, IRCCS. The main results of the clinical trial will be published in a peer-reviewed scientific journal. The final publication will be written by one of the Investigators on the basis of the final analysis performed by the Fondazione Policlinico Universitario Agostino Gemelli, IRCCS. All publications, abstract or presentations including data related to the present trial will be submitted for review to the PI prior to submission. Publication will be done in case of positive study results as well as negative results.

## Discussion

The theory of limited diffusion of antiblastic drugs from systemic circulation into peritoneal cancer implants, due to the plasma-peritoneal barrier [25], supports combining intravenous and intraperitoneal chemotherapy. Intravenous chemotherapy targets systemic metastasis and accumulates antiblastic agents in the subperitoneal space, while intra-abdominal administration allows penetration into peritoneal nodules from the peritoneal side. Moreover, systemic drug uptake from the peritoneal cavity targets the liver due to the first-hepatic passage.

This approach seems rational, especially for pancreatic cancer, which often spreads to the liver and peritoneum. However, its efficacy lacks solid scientific evidence. This research aims to provide reliable data to design future controlled trials to determine if this combination is clinically advantageous for pancreatic cancer patients.

Two recent Asian trials combined systemic Gemcitabine-Nabpaclitaxel with intraperitoneal solvent-based paclitaxel, yielding encouraging results [9, 26]. Takahara et al. conducted a phase I study to determine the recommended dose of systemic Nabpaclitaxel-Gemcitabine and intraperitoneal solvent-based Paclitaxel given through an implanted peritoneal-access port on days 1, 8, and 15 every 28 days. The doses identified were 30 mg/m^2^ of intraperitoneal Paclitaxel, 1,000 mg/m^2^ of systemic Gemcitabine, and 125 mg/m^2^ of systemic Nabpaclitaxel. The study concluded that there was no systemic toxicity from adding intraperitoneal Paclitaxel, with no adverse events or deaths related to its administration. Major issues with intraperitoneal chemotherapy were related to port management, as 33 % of patients developed port-related complications. The response rate and disease control rates were 25 and 75 %, respectively, based on RECIST criteria.

Yamada et al. conducted a phase I/II study with the same drugs but encountered dose-limiting toxicities in three out of four patients at level 1 dose, leading to lower recommended doses compared to the previous study. They established the recommended phase II dose (RP2D) at 800-75 and 20 mg/m^2^ for intravenous Gemcitabine-Nabpaclitaxel and intraperitoneal Paclitaxel, respectively. In the subsequent phase II study on 46 patients, the response and disease control rates were 49 and 95 %, respectively.

In this trial, we combined systemic Nabpaclitaxel-Gemcitabine with intraperitoneal Nabpaclitaxel administered through PIPAC. PIPAC is a safe, repeatable, minimally invasive procedure that can be combined with various systemic chemotherapy regimens 27], [28], [29. Compared to port-based intraperitoneal chemotherapy, PIPAC offers deeper tissue penetration, better drug distribution within the peritoneal cavity, and no port-related complications such as infections, ascites effusion, bowel obstruction, or peritoneal adhesions. Furthermore, it allows repetitive evaluation of the abdominal cavity and PM tissue sampling.

Five published studies on PIPAC for pancreatic cancer PM documented its safety and ability to induce pathological tumor regression on pancreatic peritoneal metastases. Four used PIPAC with cisplatin-doxorubicin as monotherapy in a salvage setting 13], [14], [15], [16, and one combined PIPAC-cisplatin-doxorubicin or PIPAC-oxaliplatin with various systemic regimens [17].

The choice of Nabpaclitaxel for PIPAC was based on its documented activity against pancreatic cancer cells and its suitability for intraperitoneal administration [30]. Cristea et al. demonstrated in a phase I study that IP administration of Nabpaclitaxel has a favorable toxicity profile, significant pharmacologic advantages, and promising clinical activity [31]. From a pharmacokinetic perspective, besides its favorable molecular size, albumin presence enhances tumor drug penetration. Both stromal fibroblast and pancreatic tumor epithelial cells exhibit high levels of SPARC, promoting Paclitaxel delivery inside pancreatic cancer cells [32]. Additionally, the high pressure of aerosolized drugs in the PIPAC procedure can overcome the high interstitial pressure in tumor tissue, critical for pancreatic cancer pharmacokinetics.

A dose-escalation study explored the safety of IP Nabpaclitaxel and determined its RP2D for PIPAC at 140 mg/m^2^ [21]. The most frequent treatment-related toxicities were liver toxicity (75 %) and anemia (70 %). Forty percent of patients at the highest dose had surgical wound infection or dehiscence. Hematological toxicity was moderate, with one patient developing grade 3 neutropenia. No grade 4 or 5 morbidity was reported. The authors concluded that PIPAC with Nabpaclitaxel was well-tolerated. Considering no DLT was observed, the MTD and RP2D were defined as 140 mg/m^2^, with an advised RP2D of 112.5 mg/m^2^ for patients with hepatobiliary impairment. Thirteen patients combined PIPAC with systemic chemotherapy, though none received taxane-based systemic regimens. PIPAC was administered every four weeks.

PIPAC has been successfully combined with several intravenous regimens [33]. A recent review concluded that combining systemic chemotherapy with PIPAC is feasible [11]. The regimen under investigation comprises six systemic cycles (according to the standard regimen [34]), each consisting of three administrations of Gemcitabine-Nabpaclitaxel on days 1, 8, and 15 plus three Nabpaclitaxel-PIPACs, one every two systemic cycles.

To reduce toxicity risk, we set the PIPAC Nabpaclitaxel dose at 112.5 mg/m^2^, lower than the 140 mg/m^2^ recommended by the Ghent group. PIPAC cycles are spaced every eight weeks, with PIPAC application 13–15 days after the last systemic administration and chemotherapy resumption 7–10 days after PIPAC. A dose level reduction of systemic chemotherapy according to EMA is also planned.

We expect the combined systemic/intraperitoneal treatment to be feasible, safe, and well-tolerated. The primary endpoint, disease control rate (DCR), will assess antitumor activity. Despite the challenges of assessing PM through RECIST criteria, DCR, comprising complete responses, partial responses, and stable diseases, should reflect the combined treatment’s activity. Histologic tumor regression and visual macroscopic disease evaluation by laparoscopy are secondary endpoints.

The main weakness of this trial is the lack of a preliminary phase I study assessing the drug combination. However, dose-escalation trials have documented the feasibility and safety of combining systemic Nabpaclitaxel-Gemcitabine with intraperitoneal solvent-based Paclitaxel [9, 26]. The well-established Nabpaclitaxel-Gemcitabine regimen for metastatic pancreatic cancer includes defined dose reduction steps [34]. Celeen et al. documented that PIPAC Nabpaclitaxel results in a median PIPAC total PTX plasma Cmax and AUC 0–24 h 2.5 times lower compared to catheter-based Nabpaclitaxel IP delivery, suggesting better peritoneal tissue drug uptake with less systemic exposure [21]. This pharmacokinetic data supports the combined intraperitoneal/intravenous treatment’s availability.

After 10–13 days from the last intravenous Nabpaclitaxel administration, Paclitaxel should be cleared, avoiding overdose following PIPAC administration. Safety will be closely monitored with dose adjustments for subsequent PIPAC cycles if toxicity occurs. Simon’s two-stage design allows stopping the study for futility if seven or more patients out of twelve demonstrate disease progression in the first stage.

Despite the pancreas being a retroperitoneal organ, pancreatic cancer frequently spreads to the peritoneum, requiring adequate treatment. This research addresses these needs. If results support adding Nabpaclitaxel-PIPAC to standard systemic treatment for metastatic pancreatic cancer, further controlled trials can be planned.
